# A deep learning approach to gender equality: Forecasting educational indicators with 1D-CNN aligned with SDG 5

**DOI:** 10.1371/journal.pone.0332273

**Published:** 2025-09-16

**Authors:** Ghada Alturif, Alaa A. El-Bary, Radwa Ahmed Osman

**Affiliations:** 1 Department of Social Work, College of Humanities and Social Sciences, Princess Nourah bint Abdulrahman University, Riyadh, Saudi Arabia; 2 College of Engineering, Basic and Applied Science Institute, Arab Academy for Science, Technology and Maritime Transport, Alexandria, Egypt; 3 National Committee for Mathematics, Academy of Scientific Research and Technology, Cairo, Egypt; 4 Council of Future Studies and Risk Management, Academy of Scientific Research and Technology, Cairo, Egypt; University of Coimbra: Universidade de Coimbra, PORTUGAL

## Abstract

Sustainable development goal (SDG) 5 focuses on gender equality and empowerment and it is considered as one of the most important SDGs. Therefore, this article presented a time series prediction model that predicts gender-related educational results in the US, Saudi Arabia, China, Egypt, and Sweden. By analyzing gender-disaggregated demographic, socioeconomic, and educational data, the 1 DCNN can reveal temporal patterns and discrepancies. The main reason for selecting 1D-CNN as a deep learning model is its ability to model sequential data and detect minor changes. Through implementing the 1 DCNN with verified historical data, realistic progress trajectories have been predicted, which are suited to the particular circumstances of each country. The results obtained from the proposed model show that the model can produce important predictions in a range of gender-focused educational measures. In addition, it provides useful information that helps organizations develop, educators, politicians, and gender activists. In Conclusion, the results presented in this paper improve evidence-based planning and focused interventions, which hasten the advancement of gender equity in education and other fields.

## 1 Introduction

**Fig 1:**
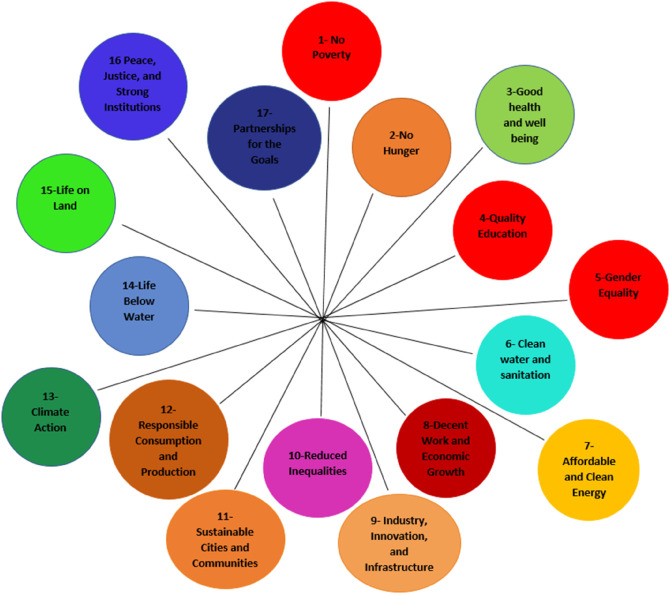
Sustainable development goals (SDGs).

To create a more sustainable and equitable future, a worldwide commitment by 193 member states was made when the UN General Assembly approved the 2030 Agenda for Sustainable Development in September 2015 [1]. This agenda includes 17 Sustainable Development Goals (SDGs) to address the most important global issues as shown in Fig 1, 169 goals with 244 indicators. SDG 5 is one of the most important SDG, SDG 5 aims to empower women and girls all over the world and achieve gender equality. Among all the 17 SDGs, SDG 5 plays an important role in achieving the goal of the 2030 Agenda [2]. Ending gender-based violence, ending harmful practices like child marriage and female genital mutilation, giving women equal participation and leadership opportunities, and ensuring universal access to sexual and reproductive health and rights are the main targets of SDG 5. It has nine targets and fourteen indicators [3]. In order to provide women equal access to financial resources, land, inheritance, property ownership, and natural resources, it also promotes structural changes [4].

One of the key element of SDG5 is redistributing and recognizing unpaid care and family labor through enhanced public services, infrastructure, social safety programs, and the encouragement of shared responsibility within homes. It also helps how enabling technologies help to foster girl’s and women’s empowerment. The SDGs 1 (No Poverty), 2 (Zero Hunger), 3 (Good Health and Well-Being), 4 (Quality Education), and 10 (Reduced Inequalities) all these SDGs have indicators related to women’s empowerment and quality of life, which make them important intersections with gender equality [5]. The importance of achieving SDG 5 has been discussed through different resources and research to achieve the goal of 2030 Agenda [6,7]. Through partnerships, advocacy, capacity development, policy impact, and monitoring initiatives to advance gender equality and empower all women and girls, the international community plays a critical role in advancing SDG 5 [8]. To promote a more inclusive and equitable society is a top objective, rethinking on how education, information exchange, and awareness-raising may destroy gender stereotypes and eliminate systemic injustices in order [9].

Furthermore, advance gender equality has been investigated using artificial intelligence (AI), one of the most promising techniques using nowadays. The major issues that can have an impact women and affect communities are the high expense of updating digital infrastructure, cybersecurity, and data privacy [10]. The digital gap is considered as a significant obstacle, especially in areas where women couldn’t be able access to digital literacy training and technology. Furthermore, the absence of development in AI-related sectors and gender-sensitive design are a result of lack of women’s presentation. This can cause a biased algorithms that perpetuate discriminatory practices and gender stereotypes [11]. AI systems have the potential of sustaining disparities in healthcare, financial services, education, and employment decision-making if intentional action is not taken. In order to ensure that AI technologies support rather than impede women’s and girls’ empowerment in accordance with SDG 5 goals, it is imperative that these barriers be addressed [12]. SDGs like SDG 6 and SDG 11 are supported by AI, which increases efficiency in industries like environmental monitoring and building [13]. In order to inform policy, a 1D-CNN model also predicts SDG 1 trends for five countries, providing precise estimates of poverty from 2024 to 2030 [14,15].

In addition to improving global comparisons, predicting SDG outcomes across regions aids in the creation of focused gender equality policies [16] and helps pinpoint important areas that require immediate attention [17]. In this regard, SDG indicators function as thorough standards for assessing advancements in equality and female empowerment [18].The purpose of this study is to use a one-dimensional convolutional neural network (1D-CNN) model to forecast SDG 5 results. The study offers important insights into the efficacy of national policies advancing women’s rights and equality by projecting gender-related metrics for China, Egypt, Saudi Arabia, Sweden, and the US. While there are synergies with SDGs 1 (No Poverty) and 6 (Clean Water), SDG 5 (Gender Equality) in China must contend with trade-offs with SDG 13 (Climate Action). Policies at the provincial level have to be modified to take these dynamics into account [19]. Egypt uses films, animations, and celebrities to promote SDG 5 on Facebook, with a particular emphasis on women’s empowerment and topics including child marriage and abuse [20]. Furthermore, Saudi companies are changing gender norms and promoting more female labor participation under the direction of SDG 5 and Vision 2030. 28 women’s perspectives highlight the beneficial effects of these activities and show how institutional logics might coincide to promote gender equality [21]. Sweden demonstrates how regional planning may be guided by an awareness of the trade-offs and synergies between SDG goals. It emphasizes that while the majority of interactions are constructive, trade-offs must be carefully considered for balanced growth, since some have an impact on SDG 5 (Gender Equality) [22]. It is required to examine the Sustainable Development Goals in various US locations, with an emphasis on the social, economic, and environmental pillars [23]. It draws attention to regional disparities and stresses the necessity of localized approaches to advancing SDG5 (gender equality) and other SDGs.

A one-dimensional Convolutional Neural Network (1D-CNN) is presented in this paper to predict gender-related educational outcomes from 2025 to 2030 to help achieve SDG 5. The main reason for implementing the 1D-CNN model is its ability to examine temporal patterns in sequential data, which enables a thorough analysis of gender disparities in education over time. The trained data used are obtained from provided historical data from 2000 to 2022. The model includes gender-disaggregated socioeconomic and educational indicators, including female literacy rates, enrollment rates for girls, gender-based student-teacher ratios, and equal access to educational resources and digital tools. The proposed approach provides important insights into areas where gender inequalities continue to exist and areas where progress is being made by examining long-term patterns in access, quality, and performance. These projections offer a useful information for developing policies and initiatives that support educational equality, empower women and girls, and foster the achievement of SDG 5 in various country settings.

## 2 Related work

Research on gender equality, or Sustainable Development Goal 5 (SDG 5), focuses on predicting trends in fields including women’s health, education, and employment. Regression models and other analytical methods employ socioeconomic factors to forecast shifts in gender equality. Although useful, these models frequently overlook the intricate relationships that explain regional differences in gender. Achieving SDG 5 is becoming more and more dependent on more sophisticated technology that can replicate the dynamic and intricate character of progress towards gender equality. The waste recovery industry in Spain was examined in [24], which looked at SDG 5 (Gender Equality) and SDG 8 (Decent Work) compliance between 2019 and 2021. Companies struggled with SDG 5, demonstrating a decrease in women in leadership posts during COVID-19, even though they had a 61% rise in revenue and achieved SDG 8. Furthermore, according to [25], just a small percentage of instructors regularly employ gender equality measures, even though half of them do. Faculty in the humanities and women were more aware. According to the report, colleges should enhance their training and procedures to effectively include SDG 5 (gender equality).

Additionally, the European Green Deal (EGD) supports environmental SDGs like climate action (SDG 13) and clean energy (SDG 7), it ignores social challenges like poverty (SDG 1) and gender equality (SDG 5). The need for a more balanced approach to sustainability is highlighted by a machine-learning study of 74 policy papers [26]. According to [27], de-carbonization in the EU lowers inequality and enhances energy sustainability. While ambitious climate action has improved agriculture and health, it may have a detrimental effect on poverty and economic growth, necessitating corrective measures. Using the Gender Inequality Index (GII) and Global Gender Gap Index (GGI) to gauge the effect of assistance for trade (AfT) on gender inequalities, [28] examined whether AfT promotes gender equality and women’s empowerment in developing nations between 2005 and 2019. To make sure AI promotes efficient and responsible learning, further research and flexible teaching strategies were required. Moreover, with a focus on SDG 5, [29] investigated how Omani women’s entrepreneurial goals were influenced by social networking, familial support, and financial availability. It was shown that while family support and financial availability had less of an impact on entrepreneurial inclinations, social networking does.

[30] examined SDG 5 indicators in India and found a 100% correlation between crime rates and legal safeguards for women. The results aided in the comprehension of patterns of gender inequality. Furthermore, in order to evaluate the progress made towards gender equality, [31] proposed a way to aggregate SDG-5 indicators. Using proportions, it integrated six chosen indicators for India’s States and Union Territories to assess present conditions and monitor advancements or regressions, with the possibility of international comparison. Additionally, [32] looked at obstacles to SDG 5 in South Africa, such as uneven opportunities and workplace discrimination. In order to attain gender equality, it needed increased institutional, financial, and political support. Furthermore, [33] assessed IPV measurement in 36 countries and discovered that physical IPV and controlling behaviors were roughly invariant, hence facilitating cross-national comparisons. It underlined that more testing of certain IPV elements is necessary in order to track SDG 5.2. Moreover, [34] investigated how boosting female involvement in Tanzania’s tanzanite mining industry may advance gender equality (SDG 5) and encourage women’s entrepreneurship, which would benefit women and the nation’s economic growth.

This study directly relates to Sustainable Development Goal 5 (SDG 5) by highlighting the pressing need for sophisticated forecasting methods that can capture the intricate dynamics included in gender-disaggregated educational data: Equality of Gender. When it comes to capturing the complex and dynamic interdependencies that shape gender gaps in schooling across many social and economic situations, traditional prediction models frequently fall short. While traditional methods could provide fundamental understandings, they usually lack the sophistication required to appropriately capture changes in gender equality, girls’ educational access, and systemic performance disparities across time. A one-dimensional Convolutional Neural Network (1D-CNN), a deep learning model that is ideal for examining time-series data, is suggested in this paper as a solution to these drawbacks. When it comes to recognizing and simulating time trends in gender-specific indicators like female enrolment rates, girls’ literacy development, and gender-based success gaps, the 1D-CNN architecture is exceptional. Forecasts produced by utilizing the 1D-CNN’s capacity to reveal nuanced and intricate patterns that conventional models frequently miss yield more precise and useful information. For policymakers, educators, and other international stakeholders striving to promote the larger goals of SDG 5 and hasten the transition to gender parity in education, these updated forecasts provide insightful advice.

## 3 Proposed model

In order to achieve Sustainable Development Goal 5 (SDG 5): Gender Equality, this section describes the creation of a prediction model intended to forecast important gender-related educational metrics. A one-dimensional Convolutional Neural Network (1D-CNN) architecture is built, gender-disaggregated data is prepared, and sophisticated machine learning techniques are used to train the model. With an emphasis on gender differences, this method makes it possible to identify intricate temporal patterns and dynamic correlations in socioeconomic, demographic, and educational information. The proposed approach creates effective prediction for the five selected countries to show their progress towards providing women and girls with equal access to education. This involves identifying these complex trends by providing data-driven insights to stakeholders and policymakers, this framework facilitates the development of focused interventions and policies that support inclusive, gender-responsive educational institutions and foster the achievement of SDG 5 goals.

### 3.1 Data collection and preprocessing

The dataset used in this study represents data collected from 2000 to 2022, this data came from the Sustainable Development Goals (SDG) Index, which is openly accessible on Kaggle [35]. This extensive dataset presents insightful information about all the countries and their international progress towards the 17 Sustainable Development Goals (SDGs) set out by the UN. This public dataset don’t contain any personal identifiable information, therefore, there is no need for ethical approval. Additionally, we recognize that the use of demographic and educational metrics, especially across various countries, has significant ethical concerns. First, results must be interpreted carefully to avoid reinforcing prejudices or failing to consider local factors. Second, while the information is aggregated at the nation level, differences in gender equality indicators may represent systemic social, cultural, and policy variables that need careful consideration. Third, cross-country comparisons must be assessed in light of varying data collecting techniques and socioeconomic situations, ensuring that findings are reported responsibly and objectively. We considered these factors throughout the research and debate, with the goal of creating conclusions that promote productive, inclusive, and culturally sensitive policy conversation.

With a focus on gender-disaggregated educational indicators like female literacy rates, girls’ school enrollment, gender parity in teacher-student ratios, girls’ access to early childhood education, and government investment in gender-inclusive educational infrastructure, special attention is paid to SDG 5: Gender Equality for this study. These metrics aid in highlighting patterns and changing differences in gender-based educational attainment and access across nations. The dataset had been assessed, and it can be found that found that there are minimal missing values, primarily in earlier years for some countries. To handle missing values, a linear interpolation technique has been employed where feasible and used mean imputation for variables with sparse missing data. To maintain data integrity, conflicting items were identified using Z-scores, and outliers were manually confirmed against national reports or other sources.

The study uses sophisticated machine learning techniques, most notably a one-dimensional Convolutional Neural Network (1D-CNN), to predict gender-specific educational paths. This approach enables precise and nuanced predictions by effectively detecting complicated temporal linkages and sequential dependencies in the data. Prior to training the 1D-CNN, a great deal of preprocessing was done, including normalizing all variables to a standardized range between 0 and 1 using MinMax scaling. In order to achieve balanced learning across gender-focused measures and account for differences in SDG scores between nations, this step was essential. The MinMaxScaler function from the sklearn package is implemented to achieve the normalization. Finally, by enhancing model performance and prediction accuracy, this preprocessing supports evidence-based policy initiatives that advance gender equality in education in accordance with SDG 5 and makes it possible to provide more accurate gender-focused educational predictions.

### 3.2 Feature selection

Overall index ratings for each of the 17 Sustainable Development Goals (SDGs) were utilized in this research, with a focus on SDG 5 (Gender Equality). In line with the UN’s goal of attaining equal opportunities for women and girls, this large dataset is a crucial tool for evaluating and forecasting progress towards gender parity in education. The growth of female literacy, gender parity in early childhood education and school enrolment, girls’ access to high-quality instruction, and the ability of educational institutions to adapt to gender-based requirements and disparities are among the main topics covered. The data is organized into a number of important columns, each of which offers insightful analysis on gender equality in educational systems:

- Country Code: To ensure uniform data administration and precise cross-country comparisons, each nation is given a distinct alphanumeric identification. This categorization scheme is essential for carrying out in-depth regional and international studies of gender gaps in education and assessing the effects of policies that support SDG 5.
- Country: The names of participating nations are listed in this column, which aids in placing gender-based educational statistics in particular geographical, cultural, and sociopolitical contexts. Gender equality in educational access and results is greatly influenced by variations in country education systems, societal norms, and governmental policies.
- Year: This component allows time-series analysis to monitor the advancement of gender parity over time by providing a temporal marker that indicates the year for each record. The influence of global events (such health crises or policy changes) on women’s and girls’ access to education is also supported, as is the assessment of the efficacy of gender-sensitive educational programs.
- SDG Index Score: This composite score provides a high-level perspective on sustainable development, including elements related to gender equity, and represents a nation’s overall performance across all 17 SDGs. It makes it easier to compare nations both within and between regions to determine which are making progress or falling behind in reaching gender equality objectives, particularly in the field of education.
- Goal 5 Score: This score assesses country efforts to achieve SDG 5 in particular. It encompasses a range of gender-related metrics, including female enrolment rates, reading levels, teacher representation, and access to inclusive, secure learning settings. The Goal 5 score offers a targeted and comprehensive evaluation of gender equality in education, pointing out areas of advancement and pinpointing areas that still require specific interventions.

The combination of the SDG Index score with the particular Goal 5 score provides context on sustainable development progress from a gendered macro and micro perspective. While the Goal 5 score provides in-depth insights into key gender equality indicators like education and political participation of women, and gender-based violence, the broader SDG Index gives a complete picture of the progress of each country, which enables scholars to evaluate the attempts of the country to counter women and gender discrimination. It is essential to uncover pertinent synergies and trade-offs in SDG 5 with other objectives like SDG 1 (No Poverty), SDG 4 (Quality Education), and SDG 10 (Reduced Inequalities) to formulate a comprehensive and integrated gender policy. The fact that the dataset is time-series adds value with respect to predictive modeling in that gender equality can be projected based on historical and longitudinal trends. For global bodies and gender advocacy NGOs, as well as state agencies working towards the 2030 gender equality goals, this makes the strategy highly instrumental. In Conclusion, this dataset is a valuable resource for evidence-based policymaking as well as a potent tool for tracking and accelerating the achievement of SDG 5, which calls for the elimination of structural impediments to gender parity and the empowerment of all women and girls.

### 3.3 Model architecture

**Fig 2:**
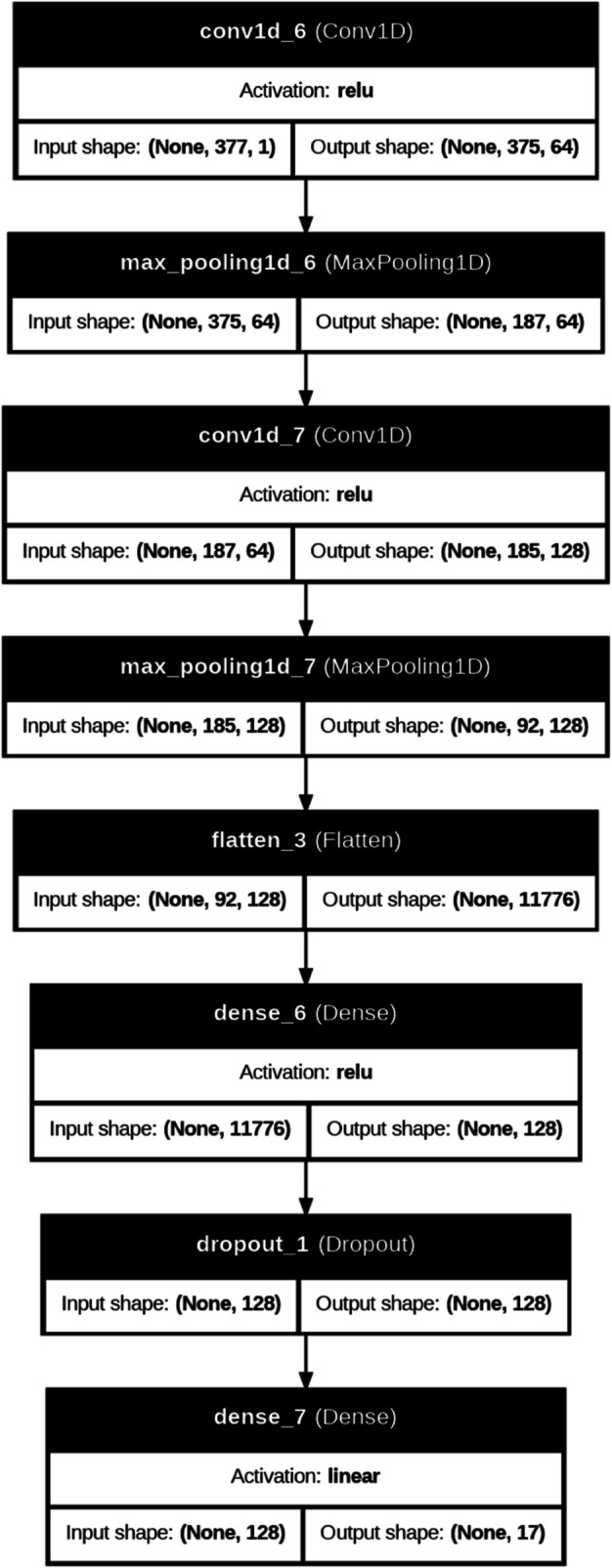
Proposed deep learning model.

A one-dimensional Convolutional Neural Network (1D-CNN) model is implemented to predict the Sustainable Development Goal 5 (Gender Equality) for five different countries. The future prediction of SDG 5 index scores and gender-specific indicators is made based on past historical data. The proposed model offers important insights into the improvements and obstacles considering gender equality. These predictions can help policymakers develop focused plans to lessen gender inequality, fight prejudice, and promote women’s and girls’ empowerment in a range of areas, including political engagement, work, education, and health. Convolutional layers, pooling layers, and fully linked dense layers make up the model architecture, as shown in Fig 2 and Table 1. These layers are all intended to extract significant characteristics and temporal patterns from time-series gender-related data. Every layer contributes significantly to the model’s increased accuracy and dependability, providing a strong foundation for comprehending further advancements in the fight for gender equality.

**Table 1:** 1D-CNN model parameters for SDG forecasting.

Parameter	Value
Time Step	3
Batch Size	50
Epochs	200
Learning rate	0.001
Dropout rate	0.5
Filters (Layer 1)	64
Filters (Layer 2)	128
Kernel Size	1
Pooling Size	2
Validation Split	0.2
Min-Max Scaling Range	(-1, 1)
Optimizer	Adam
Loss Function	Mean Squared Error
Metrics	Mean Absolute Error
Training-Testing Split	80-20
Prediction Years	2025-2030

By examining past historical data, the model is able to predict scores for SDG 5 index score. The proposed approach helps policymakers create focused initiatives to evaluate encourage women’s empowerment, and gender gaps, guarantee equal opportunities across sectors by offering insightful information on the obstacle and improvement in gender equality. All the essential components of the 1 DCNN which are convolutional layers, pooling layers, a flattening layer, and fully linked dense layers allows very accurate analysis of sequential gender-related data. The input layer receives time-series sequences made up of measures of gender equality, including women’s political representation, access to reproductive healthcare, protection from gender-based violence, labor force participation, and educational parity. To preserve temporal coherence, these sequences are organized using a predetermined number of time steps. In order to identify localized patterns and short-term variations, such as abrupt swings in labor participation or policy effect, the first convolutional layer uses several filters using the ReLU activation function. High-level temporal information are extracted by a second, deeper convolutional layer, which captures long-term trends like systematic gender prejudices or gradual advancements in women’s rights.

Max pooling layers come after each convolutional layer to lower the dimensionality while maintaining important features, increasing computing efficiency and highlighting important patterns in trends related to gender. The output is flattened, and then the thick layers learn complex interdependencies between variables, including the relationship between greater employment rates for women and girls’ education. During training, dropout regularization is used to improve model resilience and avoid overfitting. Neurones that correlate to certain SDG 5 metrics, such the proportion of women in parliament, the employment ratios between men and women, or access to healthcare, make up the output layer. These projected ratings allow for data-driven strategic planning and offer a thorough picture of a nation’s progress towards gender equality. The dataset was split into 20% for validation and 80% for training in order to construct the model. In addition to verifying the model’s predicted accuracy on unseen data, this guarantees that the model learns from a varied history dataset, including factors like gender pay inequalities, maternal mortality rates, and women’s legal rights. To assist the model avoid local minima and enhance generalization, controlled randomness was introduced during training through the use of mini-batch processing. The adaptive moment estimation (Adam) technique was used for optimization, Mean Absolute Error (MAE), and with Mean Squared Error (MSE) were the main metric used to assess model performance. Root mean squared error (RMSE) was used to get further insight into prediction variation. Whereas MAE computes the average absolute difference between real and expected hunger-related data. The coefficient of determination (${\text{R}}^{2}$) measures how accurately the model’s predictions match the actual data. It displays the ratio of variation in the dependent variable that can be predicted using the independent variables. Additionally, Mean Absolute Percentage Error (MAPE) displays the precision of forecasts in percentage terms, displaying the average absolute error compared to actual values. These metrics are critical for determining how well the model anticipates SDG 5 target results. These can be expressed as follows:

$$MSE=\frac{\sum_{j=1}^{n}(y_{j}-x_{j}{)}^{2}}{n}$$ (1)

$$RMSE=\sqrt{\frac{\sum_{j=1}^{n}(y_{j}-x_{j}{)}^{2}}{n}}$$ (2)

$$\text{MAE}=\frac{1}{n}\sum_{j=1}^{n}\left|y_{j}-x_{j}\right|$$ (3)

$$R^{2}=1-\frac{\sum_{j=1}^{n}(y_{j}-x_{j}{)}^{2}}{\sum_{j=1}^{n}(y_{j}-\bar{y}{)}^{2}}$$ (4)

$$\text{MAPE}=\frac{100\%}{n}\sum_{j=1}^{n}\left|\frac{y_{j}-x_{j}}{y_{j}}\right|$$ (5)

Where *x*_*j*_ is the projected value, *y*_*j*_ is the observed value according to educational indicators, and *n* is the total number of data. These measures aid in assessing model consistency and pointing out regions with notable prediction errors, which is crucial when spotting enduring gender disparities or postponed policy effects.

The model was trained for 200 epochs in order to avoid overfitting and guarantee pattern detection across intricate gender-related data. In each epoch, the training loop processed the whole dataset, enabling the model to constantly improve its parameters. To guarantee generalization and performance stability, training and validation losses were tracked. The proposed model predicts the indicators of SDG 5 for the next five years (2025–2030), which corresponds with the UN’s 2030 Agenda for Sustainable Development. As long as the input sequences included data between the years 2020-2022. Furthermore, the proposed model can recognize small but significant patterns, including the improvement in maternity healthcare of the increase of women in leadership positions. This approach facilitates the required decision-making based on spotting patterns and predicting future developments. Additionally, it allows stakeholders to deploy resources and carry out interventions which are urgently needed for gender-based efforts. It also acts as a diagnostic tool for assessing current gender policies and directing improved tactics to promote gender parity and sustainably empower all women and girls.

## 4 Results and discussion

**Fig 3:**
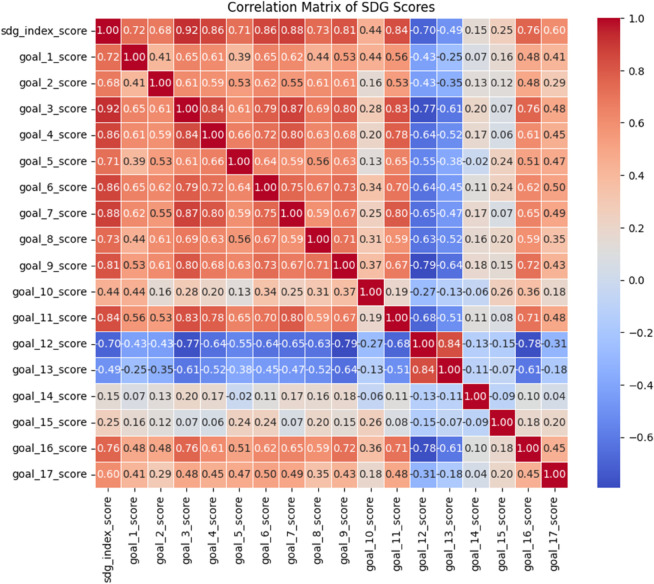
Correlation coefficients between the various SDG scores.

This section discusses the results of the one-dimensional convolutional neural network (1D-CNN) model that was used to forecast future SDG 5 outcomes. The model’s performance was assessed using both training and validation datasets, with particular attention paid to the model’s capacity to predict gender equality outcomes between 2025 and 2030 and its generalizability in a variety of gender-related settings. We explain the important conclusions from the model, analyze how these predictions may affect future gender equality policies, and compare the projected SDG 5 results with historical trends in gender-related variables. The findings are discussed in terms of real-world applications, highlighting how the model’s forecasts may direct policy choices and strategic planning meant to advance gender equality globally. We also offer a correlation chart that shows how SDG 5 and other pertinent metrics relate to one another. Fig 3 represents the correlation coefficients which quantify the strength and direction of the linear correlations between variables. Fig 3 proves the positive connection between gender equality and more general sustainable development objectives with a number of other SDGs. Policymakers may use these findings to develop plans that target many SDGs at once. Furthermore, the matrix identifies fascinating relationships that need more investigation to completely comprehend the underlying causes of these interactions.

**Fig 4:**
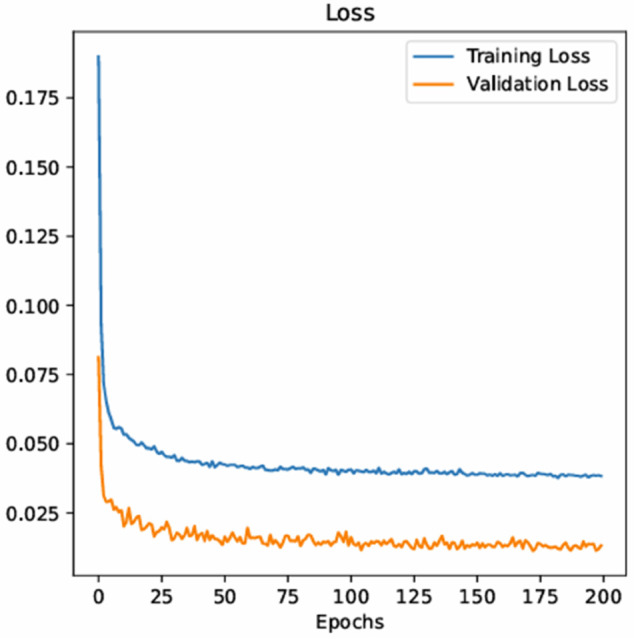
The training and validation loss curves for a 1D-CNN model.

Fig 4 displays the 1D-CNN model’s training and validation loss curves. These curves are crucial for evaluating the model’s performance and spotting any possible problems, such overfitting. The orange curve shows the model’s performance on the validation data, which is important for evaluating generalization but isn’t utilized during training, whereas the blue curve shows the model’s performance on the training data. Effective learning is shown in the early epochs by a discernible drop in both training and validation loss. The model could be overfitting to the training data, though, if the validation loss plateaus or rapidly rises while the training loss keeps down. Given that the validation loss does not significantly increase in comparison to the training loss, the curve shapes indicate that the model has attained a suitable degree of training without experiencing considerable overfitting. The model’s capacity to predict future SDG 5 (Gender Equality) outcomes is demonstrated by these loss curves. The model’s ability to generalize that is, apply knowledge from the training data to new, unseen data—is demonstrated by the consistent validation loss. The overall efficacy of the model must be evaluated through further validation using a separate test dataset. Even though the loss curves show that the model is accurately projecting SDG 5 results, more testing and improvement are required to guarantee the model’s resilience and pinpoint areas where forecasting for gender equality in the future might be strengthened.

**Fig 5:**
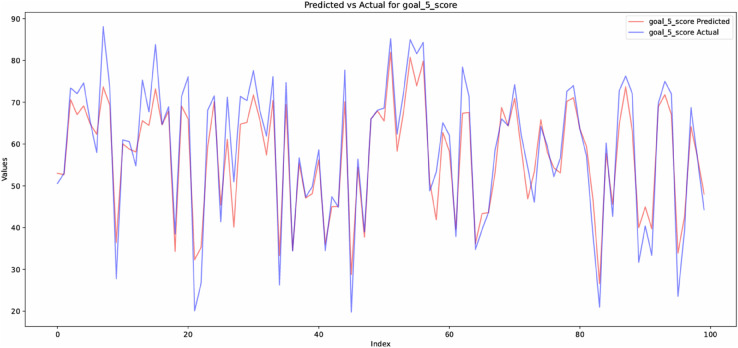
Comparison of actual and predicted SDG 5 scores.

The prediction results for Sustainable Development Goal 5 (SDG 5) scores, displayed in Fig 5, demonstrate the effectiveness of the suggested strategy. Every subplot across a variety of time steps in this figure compares the expected and actual, which is usually from 0 to 1 shows the normalization of the SDG 5 scores on the y-axis. The predicted SDG 5 scores based on the 1D-CNN model are displayed using red line, whereas the actual SDG 5 scores is represented by the blue line, either based on baseline or historical data the predication. The model’s ability to accurately forecast future gender equality results is demonstrated by the near alignment of the red (predicted) and blue (actual) lines. The model’s capacity to spot underlying trends and patterns in the SDG 5 time series is demonstrated by its precise monitoring of actual scores. Despite a few small variations, they are negligible and demonstrate the difficulties in predicting certain numbers. The majority of subplots, however, perform quite well, with little difference between the expected and actual values. By successfully capturing historical links and applying them to future forecasts, our results highlight the significance of taking time-dependent patterns into account. Additionally, this figure depicts how the proposed model can well predict SDG 5 scores, even with minor differences. Through these findings gender equality can be achieved through the well-informed decisions made by policymakers and stakeholders.

The ratings of SDG 5 for the chosen five countries (China, Saudi Arabia, Egypt, Sweden, and the US) for 2025–2030 are also included in this section. These countries were chosen because of their varied efforts to promote gender equality, socioeconomic settings, and unique methods of doing so. Through the analysis of nations from diverse geographic locations and economic backgrounds, the research aims to offer a thorough knowledge of international initiatives to advance gender equality. China has invested and put too much effort to achieve gender equality, especially for women and girls’ access to school and the job.In contrast, the United States has discovered another way to address gender inequality by providing a unique perspective through community-based programs and legislative reforms. While Sweden is the most well-known progressive country, it has implemented robust legislation to protect women’s rights and equal opportunities, particularly in the workplace and in positions of leadership. Saudi Arabia’s Vision 2030 aims to minimize gender inequities by expanding women’s participation in the workforce and education. Furthermore, Egypt is prioritizing gender equality as a developing country by aiming to reduce the gender gap in education, improve women’s access to healthcare, and increase their engagement in politics and the economy. These diverse initiatives from the chosen nations emphasize the efforts of both government and community-driven programs, as well as the great range of strategies employed throughout the world to promote gender equality.

**Fig 6:**
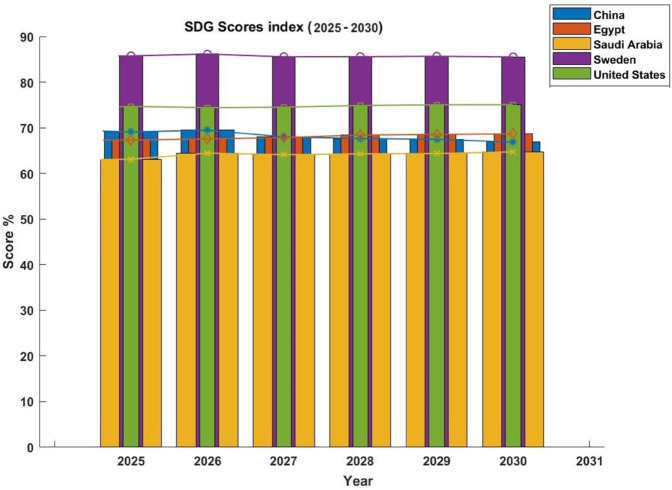
Predicted SDG index scores (2025-2030).

Fig 6 depicts the expected SDG 5 Index Scores for the chosen five nations, namely the US, Saudi Arabia, Sweden, Egypt, and China fro,m 2025 to 2030. Sweden shows its progressive efforts in achieving SDG 5 with continuously high ratings above 80%. The United States, with scores ranging from 75% to 80%, continues to make significant strides in promoting gender equality through legal reforms, women’s empowerment initiatives, and policies that encourage equal participation in the workforce and education, despite issues like gender inequality in some regions. With ratings ranging from 65% to 75%, China, Saudi Arabia, and Egypt exhibit comparable trends. By enacting laws aimed at increasing women’s and girls’ access to healthcare and education, increasing female employment, and lowering gender-based violence, these countries are significantly reducing gender disparities. Despite having somewhat lower ratings than Sweden and the US, these nations are making steady strides in important areas including equitable access to resources, boosting women’s economic engagement, and gender equality in education. This graphical comparison highlights the global differences in attaining gender equality and offers insightful information about regional possibilities and problems. It emphasizes the necessity of customized strategies that consider the unique goals and circumstances of every nation in order to guarantee gender equality for all by 2030.

**Fig 7:**
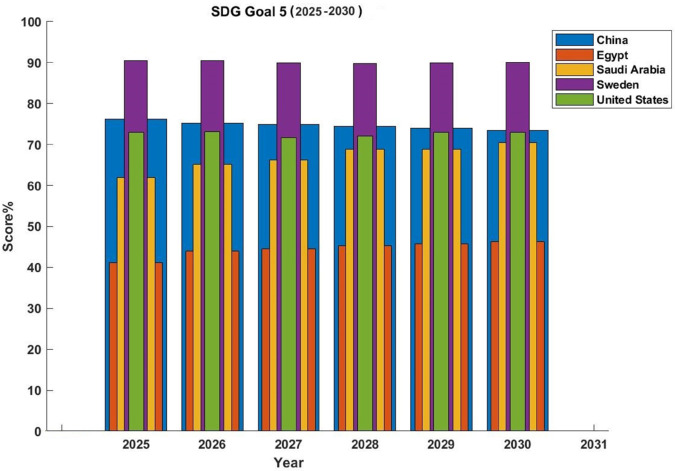
SDG goal 5 (2025-2030).

The predicted SDG Goal 5 ratings for the five chosen countries are compared and presented in Fig 7. Based on predictive algorithms applied to past SDG performance statistics, the data, shown as percentages, shows the expected development of each country towards attaining gender equality. Sweden routinely has the highest scores, approaching or staying close to 90% for the forecast period. China and The United have ratings less than Sweden but are still considered high and satisfying. This shows that these nations are in a good position to achieve their goals for gender equality, most likely as a result of continuous policy execution and ongoing investments in all-encompassing systems. Saudi Arabia, on the other hand, shows lower predicted scores, staying mostly within the 40–45% range. Additionally, it is worth be mentioned that among the five countries Egypt records the lowest value with estimations between 60% and 70%. This value shows that there is must be enduring structural and socioeconomic issues that might prevent SDG 5 goals from being fully achieved. The anticipated values’ low interannual fluctuation emphasizes how crucial long-term educational initiatives are in shaping national trajectories, as opposed to short-term interventions. This stability also emphasizes how important ongoing strategic planning is to reaching SDG goals. The observed differences across countries highlight how urgently information exchange, global cooperation, and focused policy changes are needed to help developing regions achieve gender equality.

Table 2 compares the performance of three neural architectures: 1D Convolutional Neural Network (1D-CNN), Long Short-Term Memory (LSTM), and Gated Recurrent Unit (GRU). All models were trained and evaluated using the same dataset, preprocessing processes, and early stopping criteria in consistent settings. Across all performance criteria, the 1D-CNN model outperformed both the LSTM and GRU. The proposed model has the lowest recorded Mean Squared Error (MSE) of 54.2489, Mean Absolute Error (MAE) of 6.21, and Mean Absolute Percentage Error (MAPE) of 16.46% compared with LSTM and GRU. Furthermore, the proposed model has a coefficient of determination (${\text{R}}^{2}$) with 0.8383 which is higher than the coefficient of determination of LSTM and GRU.

**Table 2:** Performance comparison of 1D-CNN, LSTM, and GRU models.

Model	MSE	RMSE	MAE	$R^{2}$	MAPE (%)
1D-CNN	54.2489	5.6158	6.21	0.8383	16.46
LSTM	345.78	18.59	14.10	0.258	51.73
GRU	300.46	17.33	12.62	0.355	46.98

The results obtained form the proposed model which refers to Gender Equality, can be used by non-technical stakeholders, such as ministries of social development, policymakers, advocacy groups, and NGOs to take a well decision for SDG 5 improvement. These results can be considered as accessible dashboards which allow stakeholders to quickly identify areas needing intervention through showing projected progress rates, gender parity gaps, and regional disparities. For example, suppose the prediction shows that gender parity in labor force participation will help increase at a slower rate than required to fulfill the objective of 2030. In that case, authorities might prioritize initiatives such as childcare subsidies, flexible working arrangements, or specialized vocational training for women. Similarly, projected stagnation in political representation may lead to the implementation of gender quotas or leadership development programs for women. The key recommendations from this paper are:

1. Legislative Support: Protecting women’s rights in political participation, education, and employment through strengthening legal frameworks
2. Economic Empowerment: The women in underserved areas should be able to access credit, entrepreneurship training, and financial resources.
3. Education and Skills Development: The representation of the female enrollment in STEM and vocational programs is currently low, then their role should be Prioritized.
4. Monitoring and Accountability: Using predicted data as benchmarks for SDG 5 performance to establish annual progress reviews.

Through simplifying the complex predictive results into new clear ones, actionable steps, the results presented in this study bridge the gap between policy formulation and data-driven analysis. The proposed approach ensures that the predicting tool serves not only academic purposes but also functions as a decision-support system which helps for achieving gender equality by 2030.

## 5 Conclusion

This study proposed a new model to predict SDG 5 for the next five years (2025-2030) for different 5 countries which are US, Egypt, Saudi Arabia and Sweden. The proposed model is built using one-dimensional Convolutional Neural Network (1D-CNN)due to its ability to model sequential data and detect minor changes. These five countries were chosen due to their varied socioeconomic backgrounds and differing strategies for advancing gender parity. The proposed model, which uses learning, is able to identify complex trends in gender-related strategic metrics from 2000 to 2022. It forecasts progress in important SDG 5 areas such female labor force participation, access to reproductive health care, gender parity in education, women’s political involvement, and the decrease of gender-based violence. The results obtained from the proposed model shows how each country will perform in the next five years to achieve SDG 5 goal. Additionally, through these prediction stakeholders and policymakers will be able to make the well-decision to improve the achievement of SDG 5. Future versions of this model may include other elements including access to economic possibilities, representation in decision-making positions, legal safeguards against discrimination, and digital inclusion. These improvements would improve the prediction power and broaden our comprehension of the complex possibilities and difficulties associated with achieving SDG 5 in various geographical areas.
